# Editorial and Miscellaneous

**Published:** 1874-06

**Authors:** 


The following pamphlets have been received: What Effect does Syphilis have upon the Duration of Life? by Fred. R. Sturgis, M.D. Electrolysis in the Treatment of Stricture of the Urethra; by Robert Newman, M.D. The Treatment of Uterine Flexions; by Ely Van DeWarker, M.D. Treatment of Vascular Nævi with the Galvanic Cautery; by B. F. Dawson, M.D., of New York.

ATLANTIC MONTHLY.—The July number of the "Atlantic" comes to hand replete with its usual feast of "good things." The Atlantic is always "tip-top," rich in contents and unexcelled in its get-up. The "Rebel" George Cary Eggleston gives its readers a few scenes behind "Confederate" times, which is good reading for summer. Price, $4. H. O. Houghton & Co., 219 Washington street, Boston.

RETIREMENT.—We learn from Dr. B. F. Dawson that he has retired from the editorial management of the *American Journal of Obstetrics and Diseases of Women and Children,* of which he was the founder. Dr. Dawson is a fine scholar and accomplished medical gentleman. The editorial brotherhood, no less than the profession, will miss his valuable services. The *Journal* is now, we learn, published by Wm. Wood & Co., N. Y.—*but we never see it.*

THE POPULAR SCIENCE MONTHLY is the best and handsomest science monthly in America—yea, in the world. It stands unrivaled—a star among all similar publications. It deals with subjects purely scientific, and is a *sine qua non* to the scientific reader of America. It is published by that always reliable publishing house, D. Appleton & Co., 549 and 551 Broadway, New York, at $5 per annum.

DEMOREST'S MONTHLY is a ladies' book of choice reading, and filled with fashion plates beautiful and attractive to all.

Eclectic Magazine.—The July number of this splendid Eclectic comes to us freighted with choice selections from the standard periodicals of the Old World. It is an indispensible monthly of art, science and literature, and should be in the hands of all reading men. Price, $5. E. R. Pelton, 108 Fulton street, New York.

Hearth and Home has changed hands, and is now published by the "Graphic Newspaper Co." It is filled to overflowing with illustrations and is a treasure-house of art. Price, $3. Address Graphic Company, 39 and 41 Park Place, New York.

Lippencott's Magazine for July is splendedly illustrated and rich in literary gems. It will be a constant source of pleasure and instruction in the family. Price, $4. J. B. Lippencott & Co., Philadelphia.

Catching Up.—We trust in the next number to fully catch up with our gleanings, which have been crowded out, to so great an extent, in the last two issues.

